# Sleep and binge eating in early adolescents: a prospective cohort study

**DOI:** 10.1007/s40519-025-01729-0

**Published:** 2025-02-26

**Authors:** Jason M. Nagata, Rachel Huynh, Priyadharshini Balasubramanian, Christopher M. Lee, Christiane K. Helmer, Kyle T. Ganson, Alexander Testa, Jinbo He, Jason M. Lavender, Orsolya Kiss, Fiona C. Baker

**Affiliations:** 1 Department of Pediatrics, University of California, 550 16th Street, 4th Floor, Box 0503, San Francisco, CA 94143 USA; 2 Factor-Inwentash Faculty of Social Work, University of Toronto, Toronto, ON Canada; 3 Department of Management, Policy and Community Health, University of Texas Health Science Center at Houston, Houston, TX USA; 4 Division of Applied Psychology, School of Humanities and Social Science, The Chinese University of Hong Kong, Shenzhen, Guangdong China; 5 Military Cardiovascular Outcomes Research Program (Micor), Department of Medicine, Uniformed Services University of the Health Sciences, Bethesda, MD USA; 6 The Metis Foundation, San Antonio, TX USA; 7 Center for Health Sciences, SRI International, Menlo Park, CA USA; 8 School of Physiology, University of the Witwatersrand, Braamfontein, , Johannesburg South Africa

**Keywords:** Sleep, Sleep disturbance, Insomnia, Eating disorder, Binge-eating disorder, Youth

## Abstract

**Purpose:**

To determine the prospective associations between sleep disturbance and binge-eating disorder and behaviors in a national sample of early adolescents in the United States (US).

**Methods:**

We analyzed prospective cohort data from the Adolescent Brain Cognitive Development Study (*N* = 9428). Logistic regression analyses were used to determine the associations between several sleep variables (e.g., overall sleep disturbance, disorders of initiating and maintaining sleep [insomnia], duration; Year 2) and binge-eating disorder and behaviors (Year 3), adjusting for sociodemographic Year 2 binge-eating covariates.

**Results:**

Overall sleep disturbance was prospectively associated with higher odds of binge-eating disorder (OR = 3.62, 95% CI 1.87–6.98) and binge-eating behaviors (OR = 1.59, 95% CI 1.17–2.16) 1 year later. Disorders of initiating and maintaining sleep were prospectively associated with higher odds of binge-eating disorder (OR = 1.12, 95% CI 1.05–1.19) and binge-eating behaviors (OR = 1.06, 95% CI 1.03–1.10). Sleep duration under 9 h was prospectively associated with greater binge-eating behaviors.

**Conclusions:**

Sleep disturbance, insomnia symptoms, and shorter sleep duration were prospectively associated with binge eating in early adolescence. Healthcare providers should consider screening for binge-eating symptoms among early adolescents with sleep disturbance.

**Level of evidence:**

Level III: Evidence obtained from well-designed cohort or case–control analytic studies.

## Introduction

Binge-eating disorder (BED) is a mental health condition marked by episodes of excessive and uncontrolled consumption of food during a brief period, usually less than 2 h [1]. It is the most common of all eating disorder phenotypes and often has its onset in adolescence. Moreover, BED is associated with significant medical and psychiatric sequelae, including type 2 diabetes, cardiovascular disease, and elevated rates of suicidality, depression, and anxiety [2, 3]. Consequently, literature has shown that BED is linked to reduced quality of life, functional impairments, and greater utilization of healthcare services [3]. Despite being more common than other eating disorders, research indicates that binge eating is often overlooked by primary care physicians in their patient evaluations, particularly in adolescents, with whom physicians have endorsed challenges discussing, managing, and screening for symptoms of binge eating [4]. While genetic, biological, socioeconomic, and psychosocial variables have been commonly investigated as risk factors for BED, health behaviors such as sleep have been underexamined as factors potentially associated with BED onset. Understanding such factors that may contribute to the onset of BED is critical for early clinical identification and prediction of adolescents who may be at higher risk of developing BED.

Among these possible factors, inadequate sleep is a widespread issue among US adolescents, with 57.8% of middle school students reporting short sleep duration in the Youth Risk Behaviors Survey (YRBS) [5]. Healthy sleep, defined as getting enough undisturbed sleep at appropriate times [6], is critical for adaptive emotional and behavioral regulation [7]. Sleep changes considerably during adolescence, driven by environmental (e.g., early school start times), psychosocial (e.g., social and peer influences), and biological (e.g., circadian phase delay) factors that promote later bedtimes while still needing to get up early for school [8, 9]. Sleep is a multi-dimensional behavior characterized by various features, including duration (amount of time asleep), latency (time it takes to fall asleep), and quality (self-satisfaction with one’s sleep). Inadequate and/or disrupted sleep can be pathognomonic of sleep disorders such as insomnia, sleep apnea, circadian rhythm sleep–wake disorders, and restless leg syndrome.

Sleep problems are strongly correlated with poorer mental health, but the relationship between poor sleep and binge eating is less well understood [10–12]. Since sleep/wake cycles are related to eating patterns [13], sleep disorders can have a detrimental impact in terms of increased disordered eating risk. For instance, children experiencing shorter sleep duration tend to consume greater amounts of hyperpalatable foods, and poorer sleep efficiency is associated with more frequent food cravings in adolescents [14, 15]. Given the additional adverse effects of poor sleep on neurocognition (e.g., decreased inhibitory control) and appetite hormones [16], further studies are warranted to understand how sleep characteristics are associated with maladaptive eating behaviors in youth, particularly certain eating pathologies like binge eating.

A *DSM-5* diagnosis of BED requires that episodes occur at least once a week for 3 months, there is an absence of regular compensatory behaviors, and there is marked distress present along with certain specified features of the episodes [17]. Binge-eating symptoms have been found to co-occur with sleep disturbances in adults and children. For example [18], US college students sleeping < 8 h a night are more likely to report binge eating, and in adult community samples, BED has been associated with greater insomnia symptoms [19]. In children, short sleep duration has been associated with an increased prevalence of eating in the absence of hunger, a common feature of binge-eating episodes [20]. Additionally, there has been literature exploring associations between sleep and loss-of-control eating in adolescents. Loss-of-control eating refers to a subjective sense of feeling out of control while eating, a behavior that is difficult to differentiate from and likely closely linked with binge eating [21–23]. One retrospective study found that variations in sleep duration and timing were associated with loss-of-control eating severity among 48 youths aged 7–18 years, while another found that night eating, loss-of-control eating, and sleep quality were interrelated across time in over 2,000 adolescents aged 11–17 years [22, 23]. However, much of the research in this area has been cross-sectional; thus, it remains unclear whether poor sleep may predict future binge-eating behaviors or BED diagnosis. Additionally, much of the literature on BED has focused on adults. A better understanding of these associations is needed in early adolescence––a critical period when health behaviors (including dietary and sleep habits) can influence psychological and medical outcomes later in life.

To address these gaps in the literature, the present investigation utilized data from a large, demographically diverse US national cohort study to examine prospective associations of sleep disturbance and related variables with BED and binge-eating behaviors in early adolescents. We hypothesized that greater overall sleep disturbance and other variables indicating poorer sleep quality would be associated with higher odds of BED and binge-eating behaviors at the 1-year follow-up.

## Methods

### Measures

We conducted a prospective analysis of data from the Adolescent Brain Cognitive Development (ABCD) Study (5.1 release, Year 2 and Year 3 follow-up). The ABCD Study is an ongoing longitudinal cohort study of brain development and child health in the US. It was established in 2016–2018 and participants were recruited from 21 recruitment sites across the US. Further details about the ABCD Study participants, recruitment, protocol, and measures are reported elsewhere [24]. At 2-year follow-up (2018–2020), most participants were 11–12 years old, and at 3-year follow-up (2019–2021), most participants were 12–13 years old. 9,428 participants met the criteria for inclusion in the study; participants without sociodemographic, depression/anxiety, binge eating, and/or sleep data were excluded. Institutional review board (IRB) approval was received from the University of California, San Diego (160,091), and the respective IRBs of each study site. Written assent was obtained from participants, and written informed consent was obtained from their caregivers.

### Independent variables

#### Sleep disturbance and characteristics

Parents/caregivers completed the 26-item Sleep Disturbance Scale for Children (SDSC) in Year 2 to assess sleep disturbance symptoms and the presence of sleep disorders among adolescents [25]. The measure focuses on the last 6 months and addresses six domains: disorders of initiating and maintaining sleep (insomnia), sleep breathing disorders, disorders of arousal nightmares, sleep–wake transition disorders, disorders of excessive somnolence, and sleep hyperhidrosis. A total score of 39 or greater indicates clinically significant disturbance [25]. We specifically analyzed the disorders of initiating and maintaining sleep subscale, since insomnia is the most common sleep disorder [26], as well as sleep duration, which the SDSC assesses by asking: “How many hours of sleep does your child get on most nights?” Possible responses were: “more than 11 h,” “9–11 h,” “8–9 h,” “7–8 h,” “5–7 h,” “less than 5 h,” or “Don’t know.” The reference range of 9–11 h was chosen, given that the American Academy of Sleep Medicine recommends 9–12 h of sleep per night for early adolescents [27]. The categories “5–7 h” and “less than 5 h” were combined given small cell sizes.

### Dependent variables

#### Binge-eating disorder (BED) and binge-eating behaviors

BED was assessed at Years 2 and 3 using the Kiddie Schedule for Affective Disorders and Schizophrenia (KSADS-5), an online tool that uses DSM-5 definitions to categorize mental health concerns in children and adolescents [17, 28]. Parents/caregivers provided reports on the frequency, duration, and features of their child’s binge-eating behaviors. BED was defined based on responses to the interview questions that corresponded to the DSM-5 criteria for BED using the KSADS-5 computerized scoring system (Online Resource 1) [28]. Binge-eating behaviors were defined as the reporting of any episode of eating more than intended and loss of control while eating in the past 2 weeks. Generally, the KSADS has demonstrated good convergent validity with clinical rating scales [28], and there is good to excellent agreement between parent and adolescent self-administered KSADS data [28]. Despite prior studies indicating low concordance between parent and child reports of binge eating [29, 30], parents are crucial reporters for eating disorders in children, as children often have less insight into their eating behaviors [24, 31].

### Covariates

Covariates were included based on their potential confounding of the association between sleep and binge eating [32]: sex (female or male), age, race/ethnicity (White, Latino, Black, Asian, Native American, or other), household income (greater than or less than $75,000, the median US household income [33]), parental education status, and study site. Since mood and anxiety symptoms may be confounders of sleep and binge eating [11, 12, 34, 35], analyses were also adjusted for a combined depression/anxiety t-score from the Child Behavior Checklist (CBCL) [36]. In addition, BED or binge-eating behavior at Year 2 (depending on the model) was included as a covariate.

### Statistical analysis

Analyses were performed using Stata 18 (StataCorp, College Station, TX). Multiple multivariable logistic regression models were used to examine the associations between sleep variables in Year 2 and BED or binge-eating behaviors in Year 3, adjusting for covariates (baseline sex, race/ethnicity, study site and Year 2 age, household income, parent education, depression/anxiety, and binge eating). Interaction by race/ethnicity was also tested to determine whether associations of sleep with BED and binge-eating behaviors differed by race/ethnicity. Sampling weights based on the American Community Survey of the US Census were applied [37]. Given multiple statistical tests, the Benjamini–Hochberg procedure was applied to adjust for the false discovery rate [38].

## Results

Table 1 describes the sociodemographic characteristics of the 9428 early adolescents included in the study. Nearly half (48.6%) of the participants were female and 43.8% were non-White. The mean age of participants was 12.0 years (standard deviation [SD] = 0.7) in Year 2. In Year 3, 0.9% of participants met the study criteria for BED and 4.1% of participants exhibited binge-eating behaviors.

**Table 1:** Sociodemographic, binge-eating, and sleep characteristics of Adolescent Brain Cognitive Development (ABCD) Study participants at Year 2 unless specified (*N* = 9428)

Sociodemographic characteristics	Mean (SD)/%
Age (years)	12.0 (0.7)
Sex assigned at birth (%) (baseline)	
Female	48.6%
Male	51.4%
Race/ethnicity (%) (baseline)	
White	56.2%
Latino/Hispanic	19.0%
Black	15.2%
Asian	5.2%
Native American	3.1%
Other	1.2%
Household income (%)	
Less than $75,000	53.4%
$75,000 and greater	46.6%
Parents' highest education (%)	
High school education or less	11.2%
College education or more	88.8%
Combined depression and anxiety t-score	53.3 (5.9)
Binge-eating disorder (Year 3)	0.9%
Binge-eating symptoms (Year 3)	4.1%
Clinically significant sleep disturbance ^a^	26.5%
Disorders of initiating and maintaining sleep subscale score (insomnia)	12.1 (3.8)
Sleep duration	
9–11 h	30.1%
8–9 h	43.7%
7–8 h	20.0%
< 7 h	6.2%

ABCD propensity weights were applied based on the American Community Survey from the US Census. SD = standard deviation

^a^Sleep Disturbance Scale for Children score ≥ 39

Table 2 shows the associations between Year 2 sleep variables and Year 3 BED and binge-eating behaviors, adjusting for covariates. Clinically significant sleep disturbance (SDSC score of 39 or greater) was prospectively associated with 262% higher odds of BED (OR = 3.62, 95% CI 1.87–6.98, *p* < 0.001) and 59% higher odds of binge-eating behaviors (OR = 1.59, 95% CI 1.17–2.16, *p* = 0.003). Additionally, insomnia symptoms (disorders of initiating and maintaining sleep) were prospectively associated with 12% higher odds of BED (OR = 1.12, 95% CI 1.05–1.19, *p* < 0.001) and 6% higher odds of binge-eating behaviors (OR = 1.06, 95% CI 1.03–1.10, *p* < 0.001). Compared to a sleep duration of 9–11 h, sleep durations of 8–9 h, 7–8 h, and less than 7 h were prospectively associated with 107% higher odds (OR = 2.07, 95% CI 1.45–2.95, *p* < 0.001), 111% higher odds (OR = 2.11, 95% CI 1.38–3.23, *p* = 0.001), and 118% higher odds (OR = 2.18, 95% CI 2.01–6.07, *p* < 0.001) of binge-eating behaviors, respectively. There was no evidence of interaction between race/ethnicity and sleep on BED and binge-eating behaviors.

**Table 2:** Prospective associations between Year 2 sleep variables and Year 3 binge-eating disorder and binge-eating behaviors in the Adolescent Brain Cognitive Development (ABCD) Study

Total sample, adjusted (a)	Binge-eating disorder		Binge-eating behaviors	
	AOR (95% CI)	*p*	AOR (95% CI)	*p*
Clinically significant sleep disturbance^a^	**3.62 (1.87, 6.98)**	** < 0.001**	**1.59 (1.17, 2.16)**	**0.003**
Disorders of initiating and maintaining sleep subscale score (insomnia)	**1.12 (1.05, 1.19)**	** < 0.001**	**1.06 (1.03, 1.10)**	** < 0.001**
Sleep duration				
9–11 h	Reference	––	Reference	––
8–9 h	1.20 (0.62, 2.31)	0.595	**2.07 (1.45, 2.95)**	** < 0.001**
7–8 h	0.90 (0.34, 2.39)	0.827	**2.11 (1.38, 3.23)**	**0.001**
< 7 h	1.78 (0.58, 5.41)	0.311	**2.18 (2.01, 6.07)**	** < 0.001**

Bold indicates statistical significance after Benjamini–Hochberg procedure. AOR = adjusted odds ratio from logistic regression models. Models represent the abbreviated output from six logistic regression models with sleep (Year 2, independent variable) and binge eating (Year 3, dependent variable), adjusted for baseline sex, race/ethnicity, study site and Year 2 age, household income, parental education, combined anxiety and depression t-score, and binge eating. ABCD propensity weights were applied based on the American Community Survey from the US Census

^a^Sleep Disturbance Scale for Children score ≥ 39

## Discussion

Using data from a US national cohort study of early adolescents (11–12 years old at Year 2), we examined prospective associations of sleep disturbance with BED and binge-eating behaviors. We found that higher overall sleep disturbance, greater insomnia symptoms, and shorter sleep duration were associated with greater odds of BED and/or binge-eating symptoms 1 year later. These findings are consistent with prior cross-sectional studies in adolescent populations that have found associations between poor sleep and binge eating [21–23].

Early adolescents who slept for fewer hours were more likely to exhibit binge-eating behaviors at the 1-year follow-up. There are several potential explanations for this relationship. First, shorter sleep duration can lead to impaired inhibitory control [39], exacerbated impulsivity in the face of negative stimuli [40], and increased perception of food as a reward [41] in adolescents. Second, poor sleep quality could be associated with nighttime binge eating, when adolescents struggle to fall asleep. A 1.5-year longitudinal study in Chinese adolescents found that poor sleep quality at baseline was related to higher night eating at follow-up (T2); this outcome was then related to higher loss of control eating at further follow-up (T3) [23]. In addition, sleep-deprived individuals [42] often experience elevated cortisol (via a hyperactive hypothalamic–pituitary–adrenal [HPA] axis in response to stress) and ghrelin [42, 43], both of which are associated with increased appetite and caloric consumption [42]. Taken together, reduced sleep may contribute to impaired control of hyperpalatable food consumption, particularly during stressful times, thus increasing the risk for BED.

Additionally, we found a significant association between clinically significant sleep disturbance with both BED and binge-eating behaviors. Sleep disturbance can lead to worse mental health outcomes, including depression, which appears to contribute to binge-eating pathology [11, 12]. Certain common features of depression, such as low self-esteem [44] and difficulties with emotion regulation and coping [35], could precipitate binge eating in adolescents. Poor sleep can also lead to decreased food-related inhibitory control [39, 45], reward system dysfunction [46], and negative affect [47], which have been shown to contribute to binge-eating episodes [35, 48].

Finally, our results showed that early adolescents experiencing insomnia symptoms had higher odds of developing BED and binge-eating behaviors at the 1-year follow-up. It is possible that insomnia in adolescents is related to the tendency to ruminate before bedtime, although our study did not specifically investigate this association. Rumination is associated with worse sleep quality, increased sleep latency, and sleep disturbances [49]. Indeed, rumination––particularly when the thoughts are eating disorder-specific (i.e., regarding weight, body shape, and eating habits)––is associated with binge eating [50] in adults with BED and obesity. Although not examined in this study, it is also possible that insomnia may be linked to nighttime engagement with social media among adolescents [51]. Increased use of social media may lead to increased shape/weight concerns and body dissatisfaction [52], which may explain the association between social media engagement and binge eating reported in prior research [53]. Insomnia due to increased rumination along with bedtime social media use, particularly when the content is related to food or body image, may play a role in binge-eating risk among early adolescents. In fact, research has shown that among US adolescents, higher screen time is prospectively associated with higher incidence of BED [54]. More research is needed to further explore the mechanisms underlying the prospective associations observed in this study.

### Strengths and limitations

The strengths of this investigation include a prospective analysis of a large, diverse cohort focused on early adolescents. Limitations should also be acknowledged. Despite adjusting for various covariates, including Year 2 binge eating and depression/anxiety, there may be other confounders that were not considered. Additionally, the potential bidirectional relationship between sleep and binge-eating disorder warrants further exploration in future research. While the prospective design represents an improvement over previous cross-sectional approaches, the observational nature of the study prevents us from establishing causality. The data utilized in this investigation were also collected during the COVID-19 pandemic, a period associated with heightened psychological stress and disruption of daily routines, which could have influenced sleep patterns and binge-eating behaviors [55]. Finally, the measures used in this study relied on both self- and parent report, which could introduce reporting bias. While parent and child reports of binge eating often show low concordance [29, 56], parents play a crucial role as reporters for eating disorders in this age group [57], as younger children may have limited insight into their eating behaviors [31]. The questions about binge eating in the current study were sourced from a reliable and validated tool, the KSADS-5, which is based on DSM-5 diagnostic criteria. Future research should prospectively examine cohorts into later adolescence, as the incidence of BED may increase. Future studies should also examine potential predictors and mediators (e.g., depression, neurocognitive functioning, social media use) of poor sleep and binge eating in early adolescent populations [58, 59].

## Conclusion

The present findings contribute to the existing literature by revealing prospective associations between sleep disturbances and BED, as well as binge-eating behaviors, among early adolescents. Overall, our results demonstrated that poor sleep predicts binge-eating psychopathology during early adolescence. These findings are particularly important given that early adolescence represents a critical developmental period, offering a window of opportunity for implementing prevention and intervention programs targeting these behaviors. Examples of interventions include providing guidelines for parents on how to foster good sleep, eating, and emotion regulation habits in children. Clinicians should be encouraged to offer counseling and anticipatory guidance to parents about the importance of sleep in the development of disinhibited/disordered eating behaviors and to collaborate with parents to address the underlying issues related to sleep or eating concerns in their children. Future research should explore the psychosocial, environmental, and biological mechanisms underlying the association between sleep patterns and binge-eating behaviors to develop more targeted interventions.

### What is already known on the subject?

Prior studies showing associations between poor sleep and binge eating have largely been cross-sectional; therefore, temporal ordering is unclear. Furthermore, associations between poor sleep and binge eating have been found in adults and older adolescents, but there is a dearth of literature focused on early adolescence, an important period for sleep and the development of eating disorders.

### What this study adds?

In a demographically diverse national sample of 9,428 early adolescents, sleep disturbance and characteristics were found to be prospectively associated with binge-eating disorder and binge-eating behaviors.

## Data Availability

Data used in the preparation of this article were obtained from the ABCD Study (https://www.abcdstudy.org), held in the NIMH Data Archive (NDA).
